# Biological determinants perpetuating the transmission dynamics of mosquito-borne flaviviruses

**DOI:** 10.1080/22221751.2023.2212812

**Published:** 2023-06-15

**Authors:** Xugang Wang, Ashraf Usama, Chen Huanchun, Shengbo Cao, Jing Ye

**Affiliations:** a State Key Laboratory of Agricultural Microbiology, Huazhong Agricultural University, Wuhan, People’s Republic of China; b Key Laboratory of Preventive Veterinary Medicine in Hubei Province, College of Veterinary Medicine, Huazhong Agricultural University, Wuhan, People’s Republic of China; c The Cooperative Innovation Center for Sustainable Pig Production, Huazhong Agricultural University, Wuhan, People’s Republic of China; d College of Veterinary Medicine, Huazhong Agricultural University, Wuhan, People’s Republic of China; e Department of Medicine, Division of Infectious Diseases, Stanford University, Stanford, CA, USA

**Keywords:** Mosquitoes, flaviviruses, determinant, vertebrate host, transmission

## Abstract

Mosquito-borne flaviviruses present a major public health concern. Their transmission is sustained in a cycle between mosquitoes and vertebrate hosts. However, the dynamicity of the virus-mosquito-host triad has not been completely understood. Herein, we discussed determinants of viral, vertebrate host, and mosquito origins that ensure virus adaptability and transmission in the natural environment. In particular, we provided insights into how proteins and RNAs of flaviviruses, blood parameters and odours of humans, and gut microbiota, saliva, and hormones of mosquitoes coordinate with each other to perpetuate the virus transmission cycle. A better knowledge of mechanisms permitting flaviviruses dissemination in nature can provide opportunities for establishing new virus-controlling strategies and could guide future epidemic and pandemic preparedness.

## Introduction

**Figure 1.**
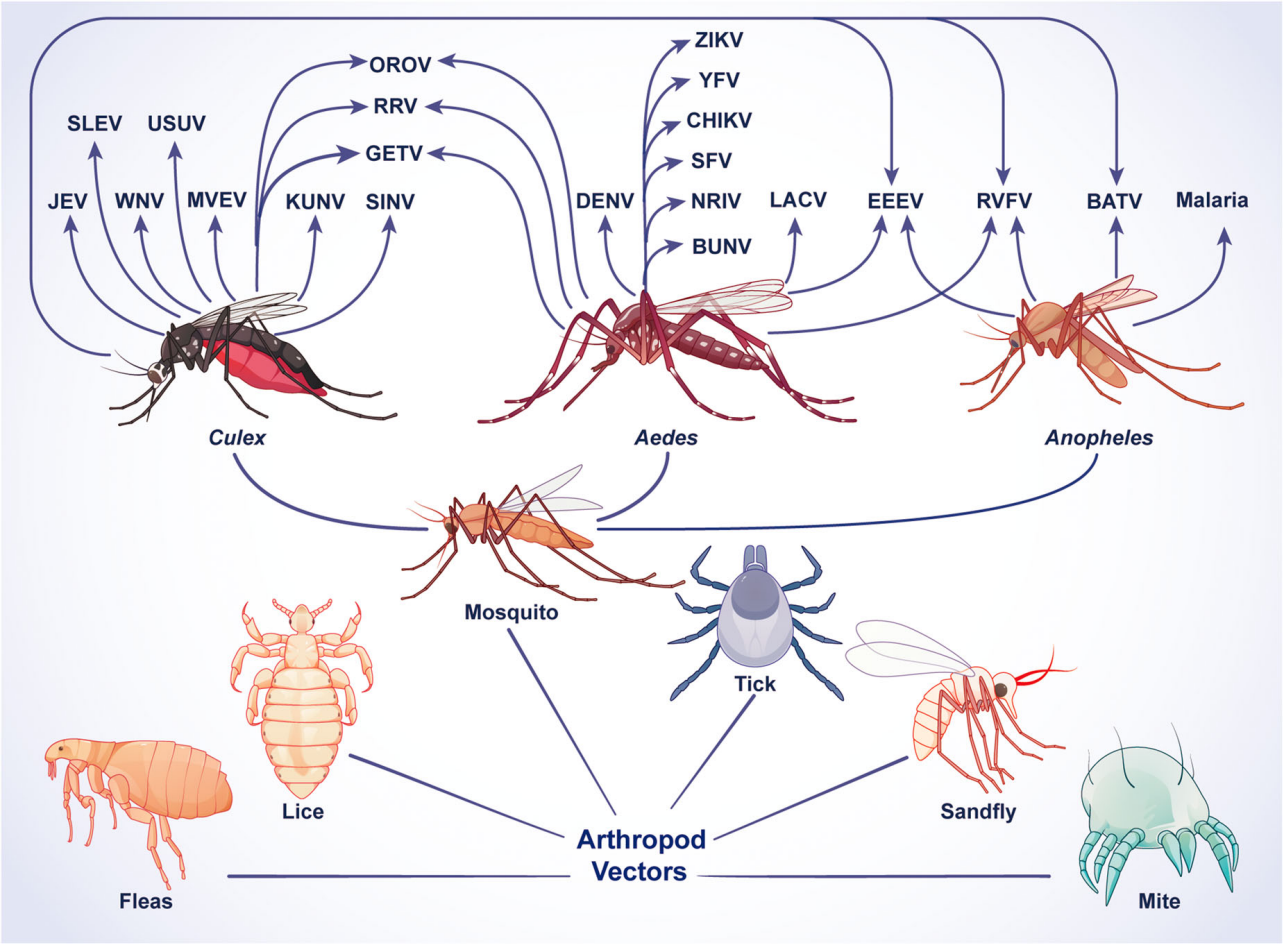
**Characteristics of arthropod vectors and the viruses transmitted by them.** Schematic showing arthropod vectors and the viruses transmitted by them. Among all arthropod vectors (mosquitoes, ticks, sandflies, mites, lice, and fleas), mosquitoes cause the most significant morbidity and mortality in humans. The viruses transmitted by specific mosquitoes are indicated. JEV, WNV, MVEV, SLEV, KUNV, USUV, DENV, ZIKV, and YFV are flaviviruses. RRV, CHIKV, GETV, SINV, SFV, and EEEV are alphaviruses. BUNV, LACV, NRIV, RVFV, OROV, and BATV are bunyaviruses. The abbreviations used are spelled out as follows: BATV, Batai virus; BUNV, Bunyamwera virus; CHIKV, Chikungunya virus; DENV, dengue virus; EEEV, Eastern equine encephalitis virus; JEV, Japanese encephalitis virus; KUNV, Kunjin virus; LACV, La Crosse virus; MVEV, Murray Valley encephalitis virus; NRIV, Ngari virus; OROV, Oropouche virus; RRV, Ross River virus; RVFV, Rift Valley fever virus; SFV, Semliki Forest virus; SINV, Sindbis virus; SLEV, St. Louis encephalitis virus; WNV, West Nile virus; YFV, yellow fever virus; ZIKV, Zika virus.

Arthropods are the primary vectors for transmitting infectious diseases, which pose a huge threat to animals and human health around the globe[1]. Among the common arthropod vectors (mosquitoes, ticks, sandflies, mites, lice, and fleas), mosquitoes cause the most significant morbidity and mortality throughout the world[2]. A total of 3 families, 38 genera, with more than 3,350 species and subspecies of mosquitoes have been recorded worldwide[3]. *Aedes*, *Culex*, and *Anopheles* are common types of mosquitoes, which can transmit flaviviruses, alphaviruses, bunyavirus, and malaria (Figure 1). Mosquito-borne flaviviruses are mainly transmitted through diverse species belonging to the genera *Culex* and *Aedes*. Among them, Japanese encephalitis virus (JEV), West Nile virus (WNV), Murray Valley encephalitis virus (MVEV), St. Louis encephalitis virus (SLEV), and Usutu virus are mainly transmitted by *Culex* mosquitoes which are characterized by spotless wings and brownish-yellow body colour, and usually, breed in various water bodies or stagnant water in containers[4, 5]. Dengue virus (DENV), Zika virus (ZIKV), and yellow fever virus (YFV) are primarily transmitted by *Aedes* mosquitoes which are characterized by black bodies with white markings, and breed in natural standing water and small containers such as pots, flower beds, bamboo tubes, tree holes, and waste tires[5, 6]. Mosquito-borne flaviviruses are responsible for a significant disease burden in humans. Clinical symptoms such as fever, headache, arthralgia, myalgia, conjunctivitis, rashes, encephalitis, neurodegeneration, neurological deficits, arthritis, and even death are shown in patients[7]. For instance, DENV alone causes ∼390 million infections annually, and 25% of subjects show serious clinical symptoms[8]. Additionally, ∼40,000 cases of ZIKV infection have been reported in 2022 in the American region and subregions according to the data from the PLISA Health Information Platform for the Americas[9]. A recent cross-sectional study conducted in the Dominican Republic has revealed birth defects and long-term neurological abnormalities in neonates born after the country-wide ZIKV outbreak in 2016-2017[10], highlighting that ZIKV-induced birth defects are still a public health concern globally.

The development of vaccines against flaviviruses remains a challenge because vaccination can either lead to severe disease due to the antibody-dependent enhancement (ADE) phenomenon or are not/less protective of other relevant serotypes/strains. Albeit there are vaccines against some flaviviruses, most of them are live-attenuated with concerns for their safety and are approved only for specific age groups under certain conditions[11]. For instance, the licensed dengue vaccine (Dengvaxia) has been approved for individuals nine through 16 years of age with previous laboratory-confirmed dengue infection and living in DENV endemic areas[12]. Considering the given circumstances, alternative strategies are required to protect from flaviviruses.

The transmission of flaviviruses is generally maintained in a cycle between mosquitoes and vertebrate hosts[13]. Flaviviruses are incidentally acquired by a naive mosquito feeding on circulating blood of a viraemic vertebrate host, and subsequently replicate to a high level in mosquito midgut epithelial cells, followed by spreading systematically to salivary glands through the hemocoel and tissue barriers [14]. Then this mosquito has capacity to transmit flaviviruses to a naive vertebrate host through the next blood meal after the viruses propagate extensively in the mosquito[15]. Previous studies have focused more on understanding the impact of flaviviruses on mammalian hosts; however, the impact of viruses on mosquitoes remained largely unknown. In recent years, several investigations have pointed-out critical factors regulating flavivirus adaptability, transmission, and epidemiological fitness. Since there are few vaccines and antivirals available against flaviviruses, this led to a sharp upsurge in their associated endemics and epidemics over the last several years. Therefore, novel insights into the virus-mosquito-host interactions governing the flavivirus transmission cycle are required.

In this review, we discussed the determinants of viral, vertebrate host, and mosquito origins, which are crucial for maintaining flavivirus transmission between mosquitoes and vertebrate hosts. Considering this knowledge, we also highlighted therapeutic strategies that could be utilized to prevent virus transmission and associated disease burdens in humans.

## Viral determinants enhancing infection and transmission of flaviviruses

**Figure 2.**
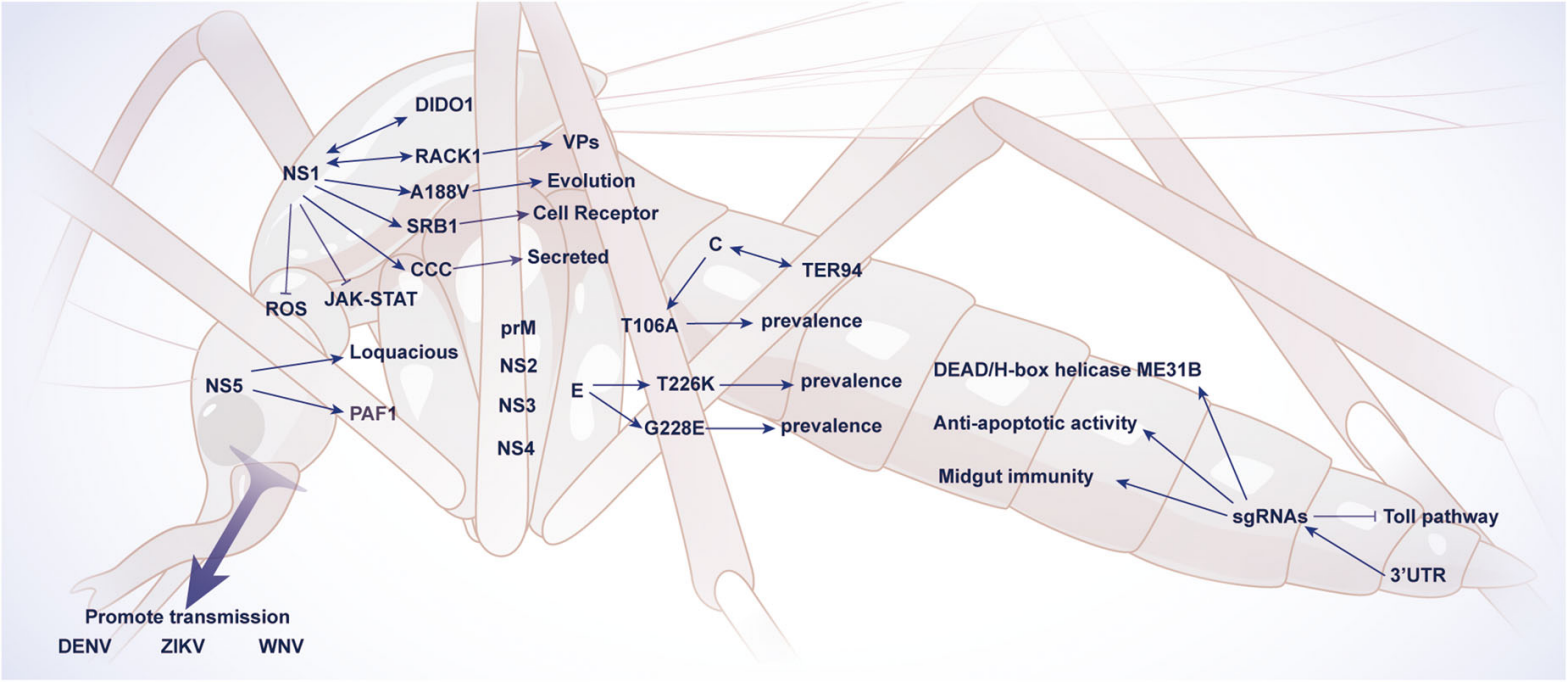
**Viral determinants enhancing infection and transmission of flaviviruses.** The interaction between viral and mosquito determinants facilitating flaviviruses replication is shown. The abbreviations used are spelled out as follows: C, capsid protein; CCC, caveolin chaperone complex; DIDO1, death inducer obliterator 1; E, envelope protein; JAK-STAT, Janus kinase signal transduction and activators of the transcription; NS, non-structural protein; prM, pre-membrane protein; RACK1, receptor for activated protein C kinase 1; ROS, reactive oxygen species; sfRNA, subgenomic flavivirus RNA; SRB1, scavenger receptor B1; TER94, transitional endoplasmic reticulum 94; UTR, untranslated regions; VPs, vesicle packets. A188V, T106A, T226K, and G228E indicate amino acid substitutions.

The evolution of flavivirus proteins and RNAs shapes virus adaptability and epidemiological fitness. Such viral variants not only impact the infection of mammalian hosts but also increase mosquito transmission, uncovering the critical determinants of virus fitness in the mosquito-mammal cycle (Figure 2). An NS1 A188V substitution in the ZIKV FSS13025 strain or American epidemic ZIKV isolates caused enhanced NS1 serum level (NS1 antigenaemia) in mice which facilitated an increased infection of *Aedes aegypti* mosquitoes while acquiring a blood meal from a viraemic mouse[16]. The evolutionary mutation T106A in the C protein of ZIKV renders the C protein a preferred substrate for the NS2B-NS3 protease which causes the maturation of viral particles. These mutant viruses showed enhanced infectivity in mosquitoes as well as mammalian hosts[17]. Similarly, the mutations T226K and G228E in the E protein of DENV2 Asian I genotype increased the binding affinity of the E protein with the mosquito C-type lectins, which resulted in enhanced viral infectivity in mosquitos and mammalian hosts, thereby promoting dengue epidemic[18]. Furthermore, the variant DENVs harbouring mutations in the 3’UTR of their genome drove the higher production of viviral subgenomic RNAs (sgRNAs) which, by disrupting the Toll innate immune response in the salivary gland, caused a higher infection rate of saliva[19]. However, the role of sequence variations in other flavivirus proteins (prM, NS2A, NS2B, NS3, NS4A, NS4B and NS5) is currently unknown.

The infection enhancement phenomenon by viral factors is also attributed by the interaction of viral proteins with the mosquito proviral factors (Figure 2). NS1, upon interaction with mosquito receptor for activated protein C kinase 1 (RACK1), promotes the biogenesis of viral replication complexes which are required for the ZIKV, DENV, and WNV replication [20]. DENV NS1 also interacts with scavenger receptor B1 (SRB1) and caveolin chaperone complex (CCC), which help virus entry and viral protein secretion in mosquito cells[21, 22]. Likewise, DENV NS1, NS3, and NS5 proteins recruit the mosquito Loquacious protein to viral replication complexes, where it binds with the viral RNAs to promote viral replication in infected mosquito cells[23, 24]. In addition, the E3 ubiquitin-protein ligase UBR5 mediates the interaction between mosquito transitional endoplasmic reticulum 94 (TER94) and ZIKV C protein, which in turn enhances ZIKV replication in mosquito cells[25]. Other mosquito proviral factors such as myosin, PI3-kinase, and myosin light chain kinase promote DENV and WNV replication in mosquito cells via interacting with their C, E, NS2A, and NS2B proteins, respectively[26].

The antagonization of mosquito immunity-related genes by viral proteins is another determinant enhancing mosquito infection and transmission (Figure 2). The DENV NS1 protein interacts with a mosquito putative epigenetic regulator death inducer obliterator 1 (DIDO1) to suppress the host antiviral responses in cultured mosquito cells, resulting in enhanced DENV replication[27]. The NS1 antigenaemia in DENV- or JEV-infected mice enhanced the virus acquisition by their corresponding mosquito vectors, which was observed through the NS1-mediated antagonization of the immunity-related genes in reactive oxygen species (ROS) and Janus kinase-signal transduction and activators of the transcription (JAK-STAT) pathways in the mosquito midgut[28]. Alternatively, ZIKV and DENV NS5 proteins antagonize immune response by blocking the recruitment of cellular polymerase-associated factor 1 (PAF1) complex[29]. Additionally, the sfRNAs of ZIKV bind with the antiviral factor DEAD/H-box helicase ME31B, which led to enhanced production of infectious mosquito saliva[30]. The WNV, deficient in sgRNA production, displayed decreased infection and transmission rates in mosquitoes when administered via a blood meal. However, such sgRNA-mutant WNVs did not affect infection and transmission when injected in the intrathoracic region, suggesting that sgRNAs participate in overcoming mosquito midgut immunity[31]. Furthermore, the ZIKV sgRNA exhibited anti-apoptotic activity in mosquito tissues as a mechanism of enhancing virus transmission by mosquitoes[32].

Overall, these studies shed light on the evolutionary adaptation of flaviviruses in mosquitoes, which could be taken into account to combat potential future flavivirus epidemics.

## Hosts determinants maintaining infection and transmission of flaviviruses

**Figure 3.**
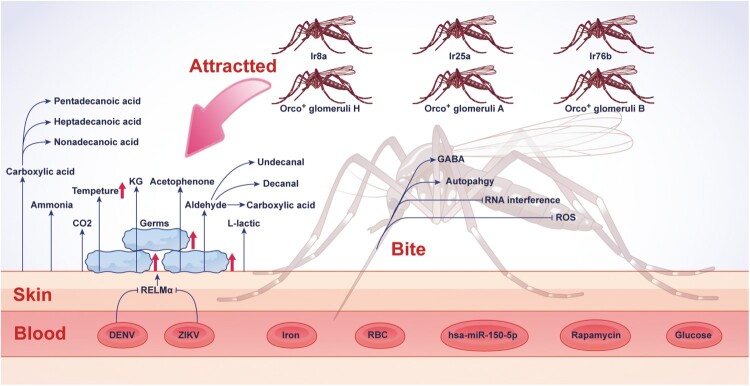
**Host determinants maintaining infection and transmission of flaviviruses.** Schematic showing human factors affecting mosquito behaviour, which contributes to virus transmission. The abbreviations used are spelled out as follows: GABA, gamma-aminobutyric acid; hsa, human; KG, ketoglutaric acid; miR, microRNA; RELMα, resistin-like molecule-α; ROS, reactive oxygen species. Orco^+^ glomeruli, Ir8, Ir25a, and Ir76b are mosquito chemosensory co-receptors.

Several host factors have been shown to affect mosquito behaviour which consequently impacts flaviviruses’ dissemination in the human population (Figure 3). Blood meal, apart from providing essential nutrients to mosquito egg development, contains elements that facilitate virus infection and spread by mosquitoes. A human-blood-derived microRNA, hsa-miR-150-5p, hijacks the mosquito Argonaute-1-mediated RNA interference system to suppress the expression of antiviral chymotrypsin that might promote flaviviral transmissibility[33]. The oral induction of glutamic acid acquired with a blood meal dampens the expression of GABAergic genes in mosquitoes which augment flaviviruses’ infection and transmission[34]. In addition, the excessive accumulation of sugar in the blood enhanced acquisition and replication of DENV in mosquitoes, suggesting that diabetic individuals are more prone to DENV infection and ultimately a cause of DENV spread by mosquitoes[35]. Conversely, serum iron in human blood activates the mosquito iron metabolism pathway to boost the activity of reactive oxygen species (ROS) in the gut epithelium, subsequently inhibiting infection by DENV[36]. Rapamycin inhibits DENV replication via the activation of the autophagy pathway in cultured *Aedes* cells[37]. Furthermore, the metabolites of flavivirus-infected hosts in the environment, such as ZIKV-contaminated urine, render mosquitoes more permissive to virus infection when breeding in the aquatic environment[38].

Human odour, heat, visual cues, and chemical substances released in the breath and on the skin also mediate the host-seeking behaviour of mosquitoes, particularly female mosquitoes. Host-seeking females prefer human odours to animal odours, suggesting the presence of unique chemicals in humans. Many studies have identified CO_2_, L-lactic acid, ammonia, short-chain carboxylic acids, long- and short-chain saturated aldehydes, and 1-octen-3-ol as human-derived attractants for female mosquitoes[39]. Of these, the mixtures of 2-ketoglutaric acid and L-lactic acid are considered the major inducers of attraction and landing for female *Aedes aegypti*[40]. The mosquito brain also harbours a human-sensitive olfactory glomerulus (Orco^+^ glomeruli H) that undergoes activation strongly upon perceiving long-chain aldehydes decanal and undecanal enriched in human odour[41]. Mechanistic findings showed that most skin-originated attractants are produced by the host skin microbiota, and fluctuation in the microbiota composition is associated with mosquito attraction to humans[42]. Moreover, ZIKV and DENV2 infection of mice promotes the proliferation of acetophenone-producing *Bacillus* bacteria on the skin of infected mice via suppressing the production of antimicrobial protein resistin-like molecule-α (RELMα), which ultimately led to enhanced mosquito attractiveness and their blood-feeding behaviour[43]. Accordingly, the dietary administration of vitamin A to infected mice caused a reduction of skin acetophenone level and hence blocked virus transmission, suggesting that the consumption of vitamin A or their derivatives， such as isotretinoin, may halt the risk of flavivirus transmission by mosquitoes in the human population[43]. Studies also unveiled reasons why some people are more prone to be bitten by mosquitoes. Mosquito neurons coexpress multiple chemosensory receptors which possibly increase the efficacy of their olfactory system[44]. Chemical analysis revealed that highly attractive people produce significantly more carboxylic acids in their skin emanations which upon sensing by mosquito chemosensory co-receptors (Ir8a, Ir25a, and Ir76b) allow mosquitoes to bite preferentially to such individuals[45]. A lack of understanding of the complexity of the mosquito olfactory system creates a hindrance in disrupting human detection by mosquitoes, which certainly requires further investigation.

Considering these studies, strategies focusing on altering the composition of host skin microbiota and establishing attractant-guided mosquito-repellent drugs may attenuate mosquito blood-feeding behaviour and may reduce the spread of mosquito-borne infections.

## Mosquito determinants regulating infection and transmission of flaviviruses

Under natural circumstances, flaviviruses are acquired by naive mosquitoes through ingesting a blood meal of an infected host, followed by extensive propagation mainly in the mosquito gut and salivary glands. Then the mosquito becomes a flavivirus reservoir and is competent to transmit the viruses to other vertebrate hosts[46].

**Figure 4.**
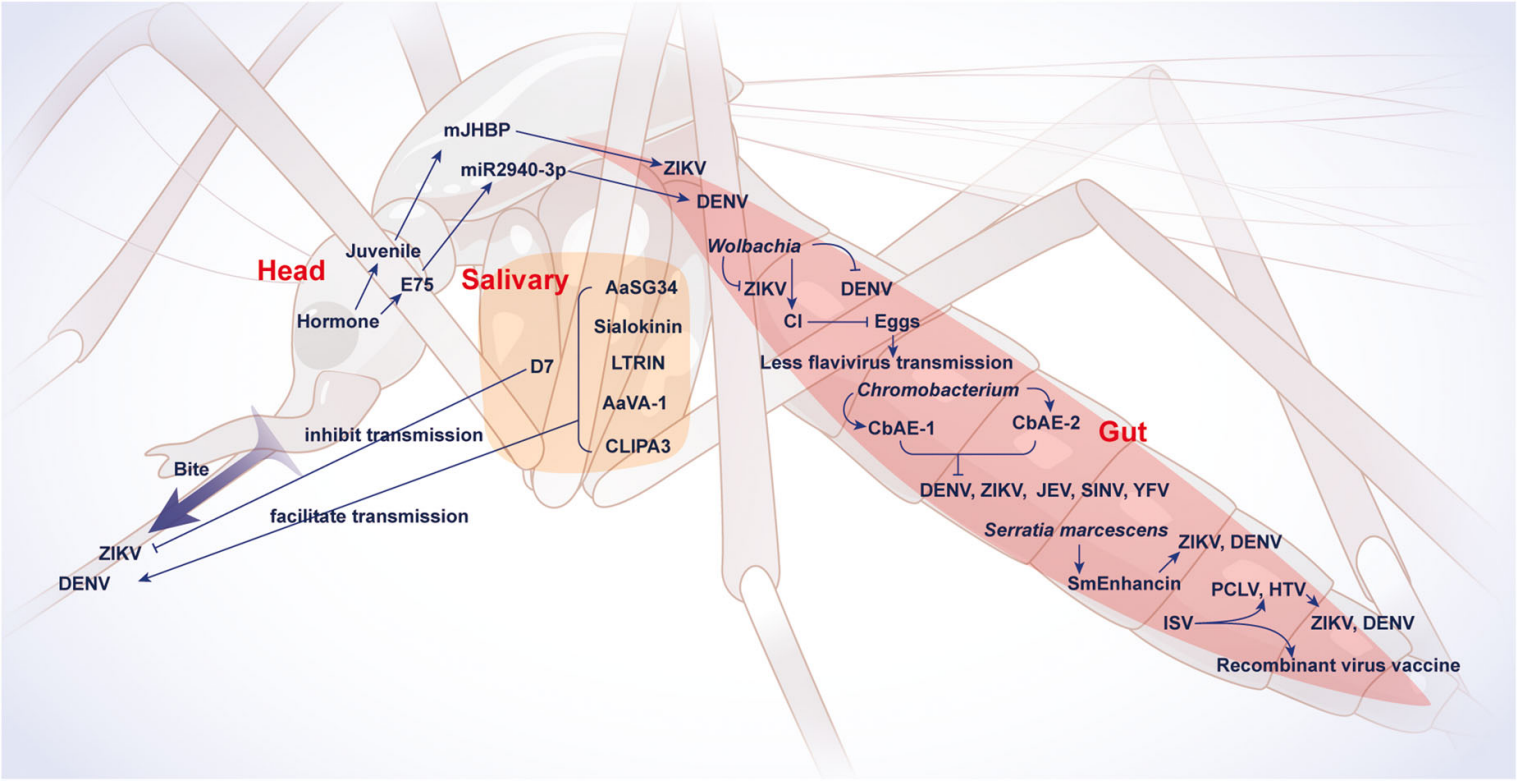
**Mosquito determinants regulating infection and transmission of flaviviruses.** An overview of mechanisms by which mosquito determinants regulate flaviviruses’ infection and transmission is shown. The abbreviations used are spelled out as follows: AaSG34, *Aedes* a*egypti* salivary gland protein of 34 kDa; AaVA-1, *Aedes aegypti* venom allergen-1; CbAE, Chromobacterium antiviral effector; CI, cytoplasmic incompatibility; DENV, dengue virus; E75, ecdysone-induced protein-75; HTV, Humaita Tubiacanga virus; ISV, Insect-specific viruses; JEV, Japanese encephalitis virus; mJHBP, mosquito juvenile hormone-binding protein; PCLV, Phasi Charoen-like virus; SINV, Sindbis virus; YFV, yellow fever virus; ZIKV, Zika virus. D7 and LTRIN are mosquito salivary proteins. CLIPA3 is a mosquito saliva serine protease.

The mosquito gut microbiota plays an important role in modulating flavivirus infection[47] (Figure 4). Among these microbes, the role of *Wolbachia* is the most well-known[48]. *Wolbachia* is an endosymbiotic, transovarially-inherited bacterium that does not normally colonize in *Aedes aegypti* mosquitoes[49]. The transinfection of mosquitoes with *Wolbachia* provides resistance to them in disseminating arboviruses, including DENV[50–52]. The increase in the *Wolbachia* density in the midgut, fatbody, and salivary gland of *Aedes aegypti* mosquitoes strongly inhibited the DENV infection in these mosquitoes[53]. The *Wolbachia*-mediated virus inhibition in mosquitoes is considered multifaceted and involves several factors, including cytoplasmic incompatibility (CI), immunity, stress, glycosylation, cell adhesion, nutritional competition, and interference in virus-mosquito cells binding[54–57]. Moreover, the inoculation of *Wolbachia pipientis* in *Aedes aegypti* mosquitoes was found efficacious in reducing the incidence of symptomatic dengue and dengue hospitalization in an Indonesian human cohort[58]. Another mosquito gut commensal bacterium, *Chromobacterium*, could be employed as an alternative to *Wolbachia*. *Chromobacterium* secretes lipases Chromobacterium antiviral effector 1 (CbAE1) and CbAE2 which demonstrated broad-spectrum virucidal activity against flaviviruses (DENV, ZIKV, and JEV) and other mosquito-borne viruses[59]. In contrast, *Serratia marcescens* promotes mosquito permissiveness to the virus via a secreted protein SmEnhancin that cleaves membrane-bound mucins, thereby facilitating DENV infection of midgut epithelial cells[60]. Hence, strategies manipulating the mosquito gut microbial flora could be established to reduce flavivirus transmission in humans belonging to other flavivirus-endemic regions of the world.

Several molecules in mosquito salivary glands and saliva are regulators of flaviviruses’ replication (Figure 4). *Aedes Aegypti* salivary gland protein of 34 kDa (AaSG34) promotes DENV2 and duck Tembusu virus replication in mosquito salivary glands possibly by dampening the expression of antiviral genes, which in turn enhanced the transmission of the viruses to the mammalian host[61,62]. The culicine-specific 34k2 salivary protein from *Aedes albopictus* (al34k2) stimulates specific IgG responses in experimentally exposed mice, indicating its immunogenicity to humans[63]. The *Aedes Aegypti* venom allergen-1 (AaVA-1) promotes DENV and ZIKV transmission by stimulating autophagy in monocytic lineage cells. This enhanced autophagy response was associated with the interaction of AaVA-1 with the leucine-rich pentatricopeptide repeat-containing protein (LRPPRC), enabling the beclin-1 to trigger downstream autophagic signalling[64]. The salivary peptide sialokinin exhibits the ability to enhance the host blood vessels’ permeability through the release of nitric oxide via the neurokinin 1 receptor (NK1R) signalling pathway, which permits the recruitment of ZIKV-permissive cells (leukocytes) at the bite site[65]. This cellular influx was sufficient to favour ZIKV replication and to worsen clinical outcomes in mice eventually[66]. Another salivary protein, called LTRIN, from *Aedes Aegypti*, enhances ZIKV transmission by binding to the lymphotoxin-β receptor to diminish canonical and non-canonical NF-κB signalling and inflammatory cytokine production in ZIKV-infected human/mouse cultured cells and in mice[67]. In addition, saliva serine protease CLIPA3 facilitates the dissemination of the DENV into the mammalian host[68]. In contrast to proviral functions, some salivary proteins are inhibitory to flavivirus infection. The D7 protein of *Aedes aegypti* has been demonstrated to inhibit DENV infection of human cultured cells and mice by directly interacting with DENV virions and recombinant DENV envelope protein[69]. An *in-silico* analysis has predicted D7 protein-derived B and T cell epitopes with affinity to predominant MHC-I[70]. In consequence, inhibiting mosquito salivary factors holds promise as a pan-viral strategy, and such proviral salivary molecules could be evaluated as potential vaccine candidates avoiding ADE to prevent virus transmission and disease in humans.

Mosquito hormone is another key factor affecting flavivirus transmission (Figure 4). A case in point is mosquito juvenile hormone (mJH), crucial for mosquito reproduction and development via regulating the ecdysone-induced protein-75 (E75) and microRNA-2940 axis[71]. In particular, the mJH-binding protein (mJHBP) is required for enhancing innate immunity and blood cell development in mosquitoes[72]. The mJH and its receptor methoprene-tolerant complex positively regulate the activity of ribosomal genes, including RpL23 and RpL27, which favours the ZIKV replication[73]. As a result, the treatment of mosquitoes with pyriproxyfen, a synthetic analog of mJH, enhanced ZIKV infection in *Aedes aegypti*[74]. These studies suggest the implementation of mosquito hormones’ analogs or antagonists in blocking flavivirus transmission.

Insect-specific viruses (ISV) also play an important role in flavivirus transmission by mosquitoes (Figure 4). The two most abundant ISVs, Phasi Charoen-like virus (PCLV) and Humaita Tubiacanga virus (HTV), increased the ability of *Aedes aegypti* mosquitoes to transmit DENV and ZIKV to a vertebrate host by blocking the downregulation of proviral histone H4[75]. The insect-specific Chaoyang virus has also been utilized as a flavivirus vaccine vector in which the pre-membrane and envelope proteins of the Chaoyang virus were replaced with those of ZIKV. The immunization with the resultant recombinant virus via mosquito bites induced herd immunity in wildlife hosts of ZIKV[76], which indicates a future avenue for mosquito vaccines to eliminate zoonotic viruses in the sylvatic cycle.

## Conclusion

Deciphering the mechanisms of how mosquitoes disseminate flaviviruses is crucial for developing controlling strategies. We systematically summarized various biological factors related to flaviviruses, hosts, and mosquitoes that maintain the transmission cycle of flaviviruses in nature. As many of these factors have been identified in recent years, further large-scale studies, dealing with such factors, are required. These studies may lay a foundation for the establishment of novel strategies to curb virus transmission and associated disease burden. In addition, tracking the evolutionary adaptations in viruses could guide future epidemic and pandemic preparedness. The strategies with a focus on modulating the vertebrate host factors, particularly body odour, seem more attractive and feasible that could be employed at a population-scale level. Similarly, harnessing the proviral mosquito salivary molecules could offer an alternative tempting approach. A recent mosquito salivary peptide vaccine (AGS-v PLUS) has been shown to induce robust broad spectrum immune responses against mosquito saliva in human subjects with no side effects[77]. Such approaches could be useful for inducing broad-spectrum protection against mosquito-borne viruses. Future studies identifying additional novel factors contributing to the virus-mosquito-host interface are required to reduce the current disease burden and to prevent future epidemics and pandemics associated with flaviviruses.

## Author contributions

WX and UA wrote the manuscript and finished the final version; WX prepared the figures; CH, CS, and YJ participated in the process of drafting and revising the manuscript. All authors reviewed, critiqued, and provided comments on the text.
